# Metallic nano-warriors: Innovations in nanoparticle-based ocular antimicrobials

**DOI:** 10.1016/j.mtbio.2024.101242

**Published:** 2024-09-14

**Authors:** Mingyou Zhang, Yuhang Cheng, Hongjin Li, Mengdie Li, Qixiang Yang, Kaifang Hua, Xiaofei Wen, Yun Han, Gang Liu, Chengchao Chu

**Affiliations:** a Xiamen University Affiliated Xiamen Eye Center, Eye Institute of Xiamen University, School of Medicine, Xiamen University, Xiamen, Fujian, China; b Fujian Provincial Key Laboratory of Ophthalmology and Visual Science, Fujian Engineering and Research Center of Eye Regenerative Medicine, Xiamen, Fujian, China; c Department of Interventional Radiology, The First Affiliated Hospital of Xiamen University, School of Medicine, Xiamen, Fujian, 361000, China; d State Key Laboratory of Physical Chemistry of Solid Surfaces & the MOE Key Laboratory of Spectrochemical Analysis & Instrumentation, College of Chemistry and Chemical Engineering, Xiamen University, Xiamen, 361002, China; e Shen Zhen Research Institute of Xiamen University, Shenzhen, 518057, China

**Keywords:** Metal nanoparticles, Ocular antibacterial, Drug delivery system

## Abstract

Eye infection is one of the most important causes of blindness. Due to the particularity of ocular structure, the enhancement of bacteria resistance, and the significant side effects of long-term medication, it is difficult to treat ocular antimicrobial diseases. The efficacy of medications currently employed is progressively becoming more restricted. The research and development of novel antimicrobial drugs is imperative and imminent in order to overcome the bottleneck problem. Metal nanoparticles have been developed rapidly in the field of biomedicine because of their brilliant antibacterial activity, long-lasting effect, and great bioavailability. Efficacy and biosafety proven in in vitro and in vivo experiments demonstrate the promising prospect of metal nanoparticles for ocular antimicrobial therapy. Based on the development status of antibacterial metal nanoparticles in ophthalmology, we summarized the antibacterial mechanism of metal nanoparticles and the application of nano-antibacterial drugs in this field, emphasizing their advantages over conventional drugs, thus guiding clinical ophthalmic antibacterial therapy.

## Graphical abstract

**Figure:**
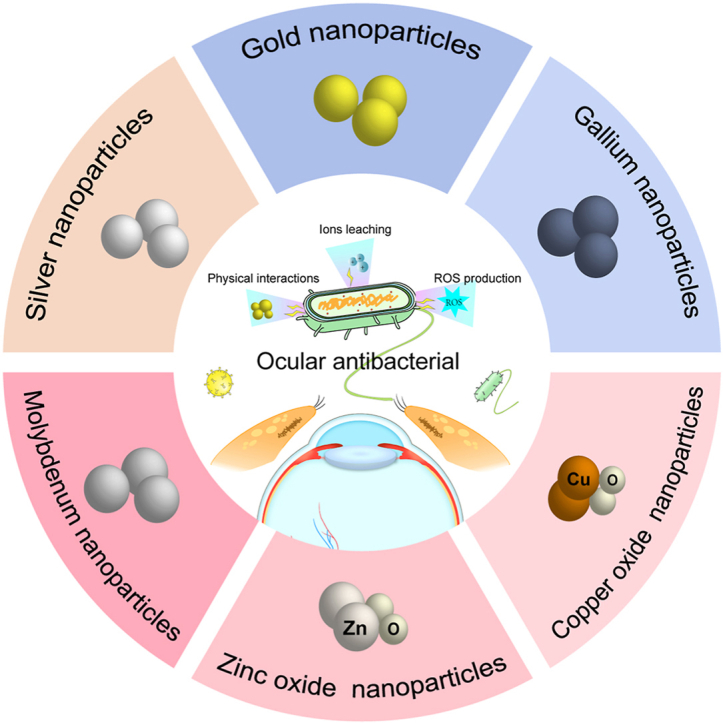

## 1 Introduction

Infectious agents, such as Gram-positive Bacteria, can cause damage to ocular infections that may lead to serious eye diseases, including conjunctivitis, blepharitis, keratitis, dacryocystitis, panophthalmitis, and even blindness [1,2]. Wearing contact lenses (CLs), experiencing trauma, using topical steroids, and undergoing surgery are significant risk factors associated with these infections [3]. In the event of a serious eye infection, prompt treatment is essential to prevent complications like corneal ulcers and blindness as the disease progresses. Bacterial keratitis, in particular, has been identified as a primary cause of corneal blindness [3,4]. Timely and effective treatment of ocular infections is crucial. However, current treatment options for these infections remain limited.

The main treatments currently available for ocular bacterial infections can be divided into drug therapy and surgery. Traditionally, topical and systemic antibiotics have been the primary drugs used clinically [5]. However, multiple anatomical barriers and drug clearance mechanisms prevent the drug from entering the infected area [6]. The hypotonic nature of the membrane and bacterial efflux systems tend to render drugs ineffective [7], and lower bioavailability impairs drug absorption, metabolism, and distribution [8]. According to research data, the concentration of antibiotics required to destroy bacteria from biofilms can be 100–1000 times higher than those required to destroy planktonic germs [9]. At the same time, different types of infection have special sensitivities to drugs, leading to difficulties in medication. The high-dose use and low bioavailability of antibiotics tend to trigger the emergence of drug-resistant bacteria. The emergence of drug-resistant bacteria further reduced the effectiveness of conventional drug treatment. Therefore, improving the efficacy of drugs and finding effective alternatives is urgent and necessary.

Surgical treatments for ocular bacterial infections vitrectomy [5], therapeutic penetrating keratoplasty [10], and conjunctival flap surgery [11]. However, due to its invasive nature, surgery is the method prone to postoperative complications. Other limitations include a shortage of corneal grafts, and poor patient compliance, which can sometimes result in visual impairment instead of improvement [12]. The high cost and complexity of operation have also prevented surgery treatment from becoming mainstream antimicrobial approaches.

Metals have long been used as antimicrobial agents due to their strong inhibitory effect against bacteria at low-concentration conditions [14]. They are used for sterilization through direct physical damage, ion release and generation of reactive oxygen species (ROS) [15]. Since metal nanoparticles have durable and effective ion release properties, they are more likely to be absorbed by cells through endocytosis and microphagocytosis, using them as antimicrobial agents appears to be very promising [16]. Compared with antibiotics, metal nanoparticle-based therapies help to obtain appropriate drug concentrations and improve permeability, bioavailability and residence time [13]. Their discriminating ability to target bacteria and mammalian cells is also a great advantage over antibiotics [14]. Therefore, metal nanoparticles could be an effective and promising treatment for eye infections [17].

**Fig. 1:**
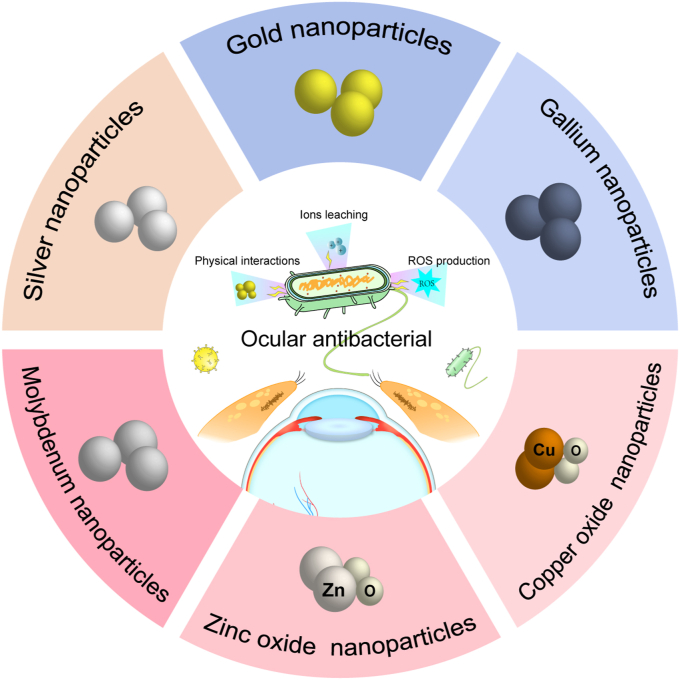
Antibacterial mechanisms of metal nanoparticles and their categorization.

In this review, we focus on Ag, Au, Ga, ZnO, Cu, MoS_2_, Ti MXenes, and Pt nanoparticles due to their demonstrated efficacy in antibacterial applications, and all of them have been put into research, particularly in ocular therapy. We classify the different metal nanoparticles, highlighting their antimicrobial mechanisms and current research progress in the field of antimicrobials. Then, we highlight the results of the existing research on different metal nanoparticles for use in ocular antimicrobials. Finally, existing challenges and future directions of metal nanoparticles in ophthalmic antibacterial applications are presented, aiming to provide new ideas for overcoming obstacles in ocular antimicrobial therapy (Fig. 1).

## 2 Silver nanoparticles (AgNPs)

**Fig. 2:**
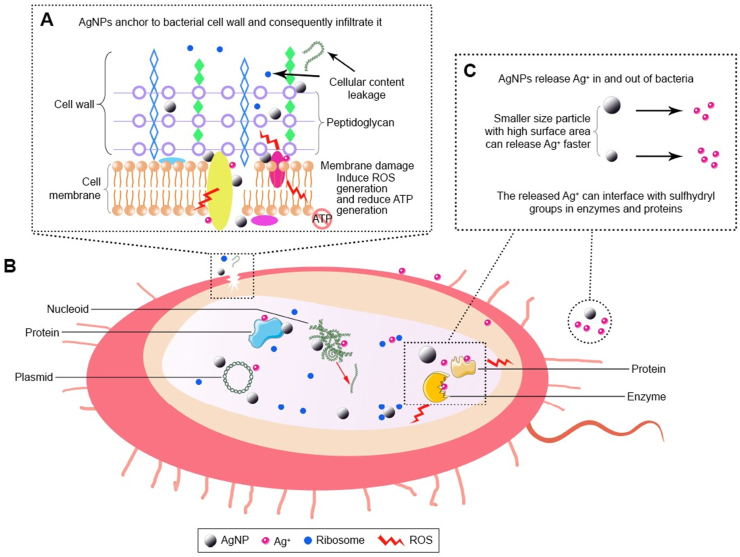
(A&B)Antbaceral mechanisms of AgNPs. Direct interactions between bacteria and AgNPs. (C)released silver ion (Ag+)-mediated antimicrobial action [23]. Reproduced with permission. Copyright 2018 Dove Medical Press Limited.

Silver nanoparticles (AgNPs) are considered the most effective metal nanoparticles against bacteria. AgNPs not only prevent the spread of drug-resistant bacteria but also exhibit strong biocompatibility and extensive antibacterial activity [18], especially spherical [19] and medium-sized AgNPs of 15.0 ± 3.6 nm [20]. Given the small size of AgNP, it is reasonable to assume that AgNPs can effectively treat intraocular infections [21]. It is known that AgNPs can disrupt membrane structure, and release ROS and silver iron [22]. Though the mechanisms can be classified into three categories—direct interaction between silver (Ag^0^) and bacteria (Fig. 2A and B), antimicrobial action mediated by released silver ion (Ag^+^) (Fig. 2C), antibacterial membrane activity of AgNPs - the exact antibacterial mechanism of AgNPs is still unclear [23]. However, due to their efficiency, which has been demonstrated by multiple studies, complex AgNPs compounds are also considered the next generation of antibiotics, as they appear to be a potential substitute for current medication drugs [24].

### 2.1 AgNPs for intraocular injections and drops

**Fig. 3:**
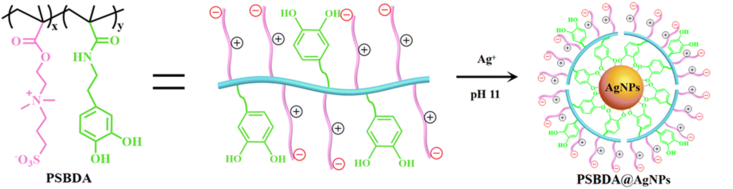
Synthesis of polyzwitterion-functionalized silver nanoparticles PSBDA@AgNPs [26]. Reproduced with permission. Copyright 2022 The Royal Society of Chemistry.

The antimicrobial effect of AgNPs on *Pseudomonas aeruginosa* (*P. aeruginosa*) and *Staphylococcus epidermidis* (*S. epidermidis*) has been confirmed in earlier studies, indicating their potential for the treatment of microbial keratitis [25]. In recent years, further studies have gradually become prominent. Xiang et al. used a copolymer, poly (sulfobetaine methacrylate-co-dopamine methacrylamide) (PSBDA), as a stabilizer and a reducing agent, to synthesize Zwitterionic PSBDA @AgNPs (ZP@Ag)(Fig. 3) [26]. The presence of zwitterionic outer layers on ZP@Ag nanoparticles contributes to the formation of a hydration layer on their surface, effectively preventing aggregation, precipitation, or elimination in the physical environment. Compared to non-zwitterionic counterparts, Zwitterionic PSBDA @AgNPs showed significantly improved antimicrobial efficiency in vitro and in vivo, as well as enhanced hemocompatibility and biocompatibility. What's more, larger-sized nanoparticles with zwitterionic coatings increase their cycling duration and successfully protect them from non-specific adsorption by proteins and microbes [27].

**Fig. 4:**
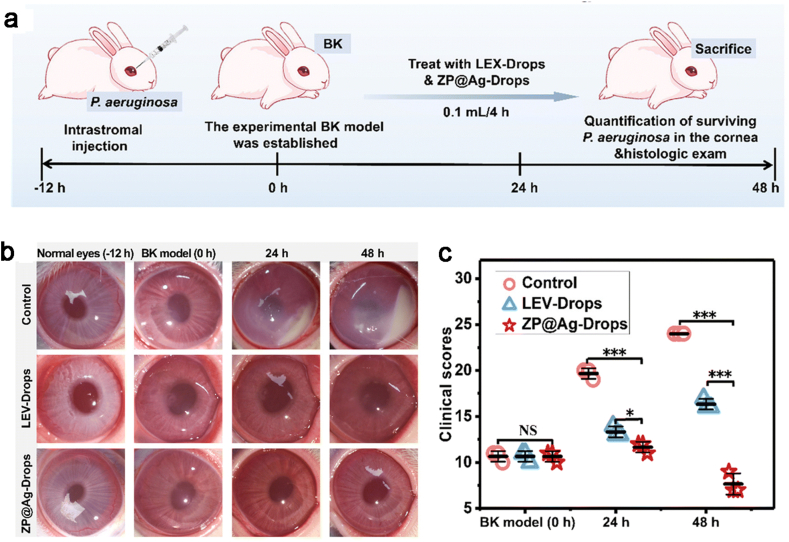
(a) Modeling and treatment of BK rabbits. (b) Slit lamp images of three groups of rabbits at 12, 0, 24 and 48 h. (c) The clinical scores of rabbit eyes after treatment for 0, 24 and 48 h [28]. Reproduced with permission. Copyright 2023 The Royal Society of Chemistry.

Based on this principle, in a recent study, Bai et al. diluted the ZP@Ag solution with phosphate-buffered saline (PBS) solution to 50 ppm to form ZP@Ag-drops and used it as a treatment for keratitis [28]. ZP@Ag-drop has been demonstrated in vitro that it has antibacterial activity against *P. aeruginosa*, *S. aureus* and Methicillin-resistant *Staphylococcus aureus* (MRSA) as well as anti-biofilm activity against *P. aeruginosa*. They created rabbit models of *P. aeruginosa*-infected keratitis and subsequently performed a series of assessments to assess the effectiveness of the treatments (Fig. 4 a). After the slit-lamp examination of rabbit eyes, the results showed that ZP@Ag-Drops were significantly effective within 48 h compared with the control groups treated with PBS solution and commercial levofloxacin eye drops (LEV-drops) (Fig. 4 b). And the difference between the groups was statistically significant (Fig. 4 c) Due to the synergistic bactericidal effect of Ag+ and reactive oxygen species (ROS), the application of ZP@Ag drops in the bacterial keratitis rabbit model showed better antimicrobial efficacy compared to LEV-drops. And it has good biocompatibility and no toxicity. This provides a new idea for the clinical treatment of bacterial keratitis.

**Fig. 5:**
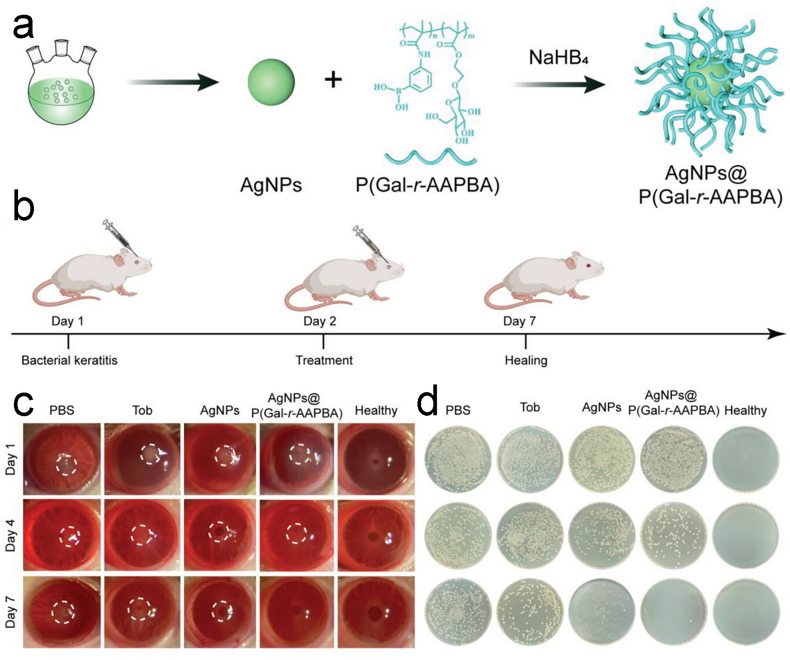
(a)synthesis of the AgNPs@P(Gal-r-AAPBA). (b)Modeling and treatment schedule of bacterial keratitis rabbits. (c)slit-lamp photographs of MDR PA-infected corneas on days 1, 4, and 7 following different treatments. (d)Bacterial culture plate of corneal secretions on the last day [29]. Reproduced with permission. Copyright 2023 Wiley-VCH GmbH.

Zhang et al. synthesized a copolymer of 3-methyl acrylamide phenylboronic acid (AAPBA) and 2-(2′,3′,4′,6′-tetra-O-acetyl-β-D-galactopyranoside) ethyl methacrylate (Gal), which they attached onto the surface of AgNPs to create AgNPs@P(Gal-r-AAPBA). (Fig. 5 a), aiming to prolong the retention time in the eyes and enhance biofilm penetration [29]. The bacteria could be assembled with copolymers, and they could be effectively killed by AgNPs. For multi-drug resistant *Pseudomonas aeruginosa* (MDRPA) and MRSA, AgNPs@P (Gal-r-AAPBA) exhibits extraordinary antibacterial efficacy and when the concentration exceeds 4.8 μg*mL^−1^, the inhibitory effect reaches 100 %, which means complete inhibition of bacterial growth. They designed the bacterial inoculation along with the therapy plan, as well as the mouse models of keratitis infected by MDRPA (Fig. 5 b). Slit-lamp photographs taken throughout therapy revealed that in contrast to the other groups, AgNPs@P(Gal-r-AAPBA) groups were able to see translucent corneas (Fig. 5 c). After counting the ocular surface secretions of the groups on the last day, it was observed that the number of bacteria decreased significantly in AgNPs@P(Gal-r-AAPBA) group, and there was also a slight decrease in the number of bacteria in the group treated with AgNPs alone (Fig. 5 d). Therefore, we can make a conclusion through this study that even AgNPs by themselves may have some antibacterial activity; however, by treating AgNPs specifically to compensate for some of their shortcomings, the antibacterial effect can be enhanced. This nanocomposite has the potential for future clinical translation, showing better ocular anti-inflammatory activity, better corneal penetration, and significantly increased drug retention on the ocular surface.

**Fig. 6:**
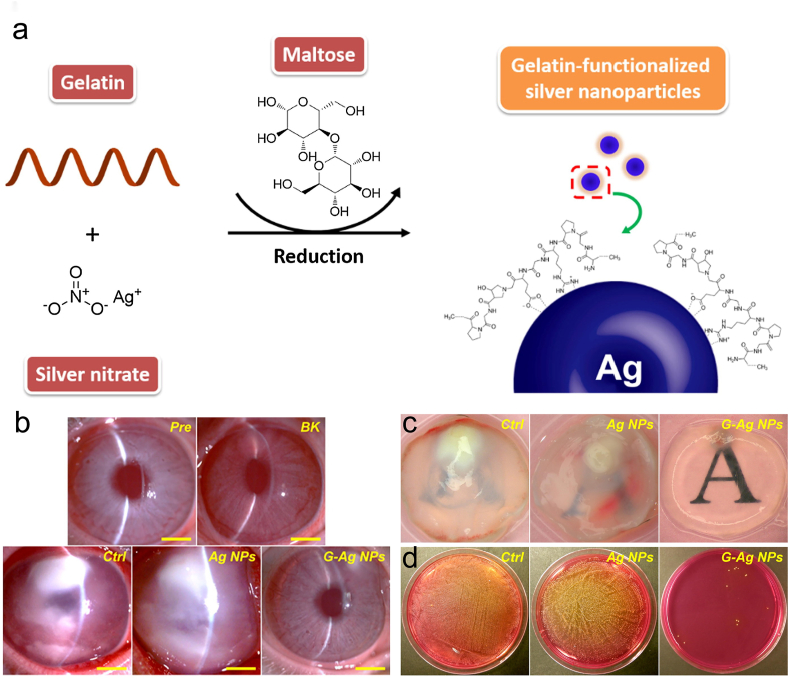
(a)The preparation process of G-Ag NPs. (b) Slit-lamp diagrams of the different groups preoperatively, three days after induced keratitis, and three days after treatment thereafter. (c) View of the word script below the corneal sample extracted after treatment. (d) Diagram of a bacterial culture plate of corneal tissue [30]. Reproduced with permission. Copyright 2018 Elsevier Inc.

In another study, Luo et al. presented the use of gelatin-capped silver nanoparticles (G-Ag NPs) as anti-infective medications to treat keratitis caused by *S. aureus* [30]. Gelatin, maltose, and silver nitrate were simply mixed to create G-Ag NPs, which had better stability and antibacterial action against *S. aureus* than uncapped Ag NPs (Fig. 6 a). G-Ag NPs will have improved biocompatibility and adhesion qualities with gelatin as a stabilizer, improving their therapeutic efficacy in reducing bacterial infections after topical ophthalmic administration. They tested the therapeutic role of G-Ag NPs in *S. aureus-induced* keratitis using an intrastromal injection model of rabbit keratitis, specifically, with the help of slit-lamp images of rabbit eyes before and after modeling, to show the therapeutic differences between the groups (Fig. 6 b). Combining the macroscopic images of corneal samples (Fig. 6 c) and bacterial culture plates of corneal tissues (Fig. 6 d), it can be clearly seen that the corneal clarity in the G-Ag NPs group was significantly improved and the antibacterial effect was superior after treatment in each group. It has significantly improved the biocompatibility, in vitro stability, and antimicrobial activity of Ag NPs by using a gelatin functionalization strategy in this study, which has great potential in the treatment of corneal bacterial infections.

### 2.2 Contact lenses loaded with AgNPs

**Fig. 7:**
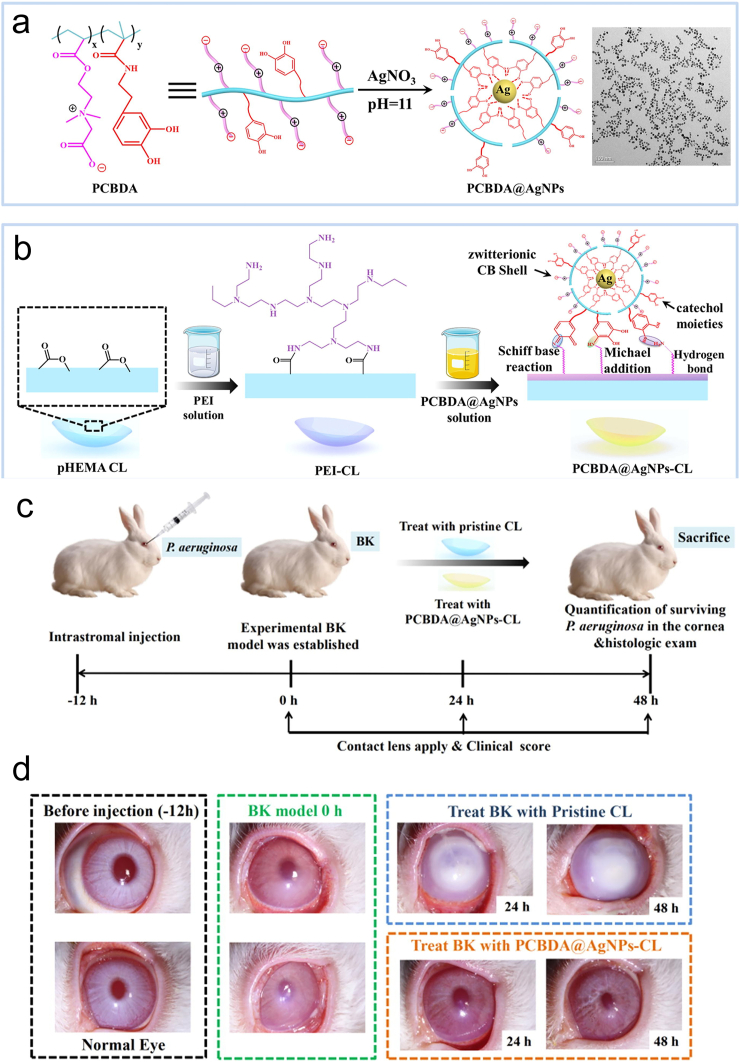
(a) The synthesis route of PCBDA@AgNPs. (b) Flowchart of PCBDA@AgNPs loaded on CLs. (c) The establishment and treatment process of BK rabbit models. (d) The photos of the eyes of BK model rabbits, normal rabbits, and rabbits treated after 24 and 48 h with PCBDA@AgNPs-CL and Pristine CL [34]. Reproduced with permission. Copyright 2021 Elsevier Inc.

In addition to treatments such as the eyedrops mentioned above, loading nanoparticles onto contact lenses to treat bacterial keratitis is a promising new approach. Since contact eyes are closely associated with corneal infections, gram-positive bacteria such as *S*. *pneumoniae* and *S. aureus*, can easily cause inflammation through contact lenses. This has led growing interest in research on antibacterial contact lenses in recent years [31]. Drug-carrying contact lenses are a novel drug-delivery technology that can continuously transfer drugs from the lens to the tear film, increase the time drugs are retained on the ocular surface and improve their bioavailability [33]. Loading metal nanodrugs on contact lenses is an effective method for antibacterial protection. It has been shown that the eye is not affected by Ag contact lens use for up to six months [32]. Taking advantage of this, AgNPs modified with zwitterionic poly (carboxy betaine-co-dopamine methacrylamide) copolymer (PCBDA@AgNPs) were created and firmly stuck onto soft CLs to develop new anti-infective therapeutics (Fig. 7 a) [34]. Then, using surface chemistry inspired by mussels, PCBDA@AgNPs are uniformly and firmly bound onto amino-functionalized pHEMA-based CLs (Fig. 7 b). This product combines the benefits of zwitterionic, AgNPs, and contact lenses to provide outstanding antifouling, antibacterial, and anti-biofilm qualities. Furthermore, in different treatment groups of rabbit keratitis models, the results showed that PCBDA@AgNPs-CL had a significant effect (Fig. 7c and d). This nano complex displayed outstanding capacity in avoiding corneal structural damage and eliminating eye diseases caused by microorganisms.

The antibacterial activity of nanomaterials is enhanced by their increased surface area and charge density, as these factors amplify the interaction forces with the surfaces of bacterial cells [35]. On this basis, positively charged Ag@PDA (polydopamine) contact lenses (Ag@PDA-2.5) were created, exhibiting outstanding efficacy in mice with bacterial keratitis [36]. Liu et al. proposed a simple and innovative technique that uses bioglue and dopamine as a reducing agent to stably bind multilayer-AgNPs to contact lens surfaces. Compared with monolayer-layer AgNP-loaded contact lenses prepared using traditional methods, the multilayer AgNP-loaded contact lenses displayed superior visible light transmittance and outstanding antibacterial activity. Additionally, in vivo therapeutic studies in mouse models have shown that Ag@PDA-2.5 has a significant therapeutic impact on *P. aeruginosa*-induced bacterial keratitis. Furthermore, there is a chance that this multilayer AgNP-loaded contact lens will be used in future combination therapy with other drugs or biomolecules.

Separately, contact lenses made of silver NP-impregnated hydrogel material showed sufficient antibacterial effects to reduce the risk of microbe-related adverse events in lens wearers [37]. Silicone-hydrogels (SiHCFs) infiltrated with AgNPs have been confirmed to inhibit bacterial growth and minimize biofilm formation, and may be used in the preparation of antimicrobial contact lenses [38]. Meretoudi et al. found that pHEMA@AgNPs (ORLE) prepared using oregano leaf extract (ORLE), AgNPs, and hydroxyethyl methacrylate (HEMA) may be a candidate for developing antimicrobial contact lenses [39]. Rossos et al. used eucalyptus leaf (ELE) and willow bark (WBE) extracts to form silver nanoparticles (AgNPs (ELE), AgNPs (WBE)) and found their antimicrobial activity against *P*. *aeruginos*, *S*. *epidermidis*, proposing their potential application in contact lenses treatment of keratitis [40]. Alarcon et al. doped AgNPs with collagen hydrogels, and the resulting composite AgNPs released silver and exhibited antibacterial activity, which is anticipated to be used as contact lenses or corneal graft implants in the future to seal ulcerated ocular surfaces [41]. A unique feature of this study is the use of different colored hydrogel lenses, which mitigated the issue of AgNPs causing intense yellow coloration. These lenses exhibited antibacterial properties equivalent to those of the antibiotic gentamicin and demonstrated biocompatibility with human corneal cells.

### 2.3 AgNPs for photothermal therapy and antimicrobial synergistic treatment

The main methods of ocular drug delivery currently available are topical infusion, subconjunctival injection, subretinal injection, and intravitreal injection [42]. The invasive techniques may lead to intraocular infections and retinal detachment as post-administration consequences. In contrast, nanoparticle delivery systems have the potential to reduce injection frequency and minimize intraocular complications [21]. Mild-temperature photothermal adjuvant therapy offers significant advantages in ocular antimicrobial therapy, as it provides antimicrobial properties while reducing heat loss to nearby tissues [43]. The synergistic antimicrobial effect of photothermal therapy using metal nanoparticles is also being studied as an important strategy.

**Fig. 8:**
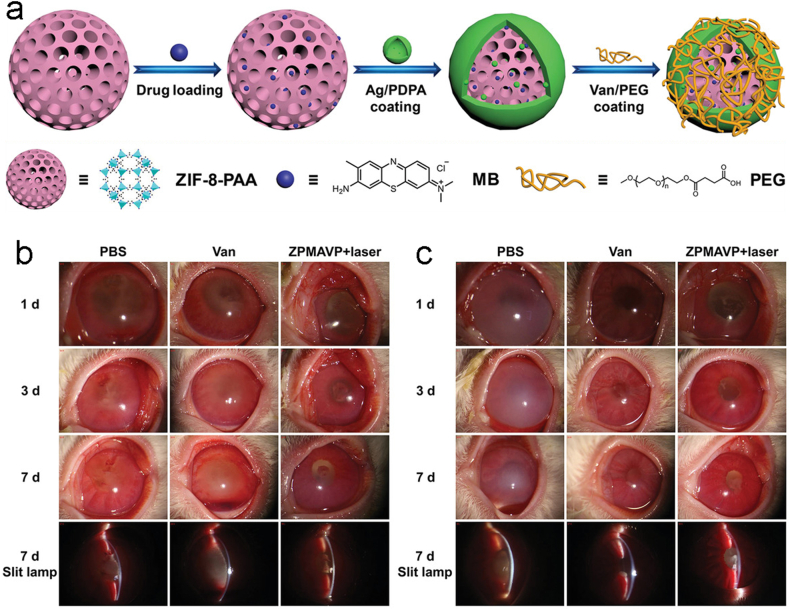
(a)The construction process of ZPMAVP. Slit-lamp micrographs and images of endophthalmitis caused by (b)*S. aureus* and (c)MRSA after varying treatments for 1, 3, and 7 days with PBS, Van, and ZPMAVP NPs + laser [44]. Reproduced with permission. Copyright 2019 WILEY-VCH Verlag GmbH & Co. KGaA, Weinheim.

Researchers have applied AgNPs to the construction of metal-organic frameworks that can form drug-delivery systems to assist in therapeutics. Chen et al. constructed a zeolitic imidazolate framework-8-polyacrylic acid (ZIF-8-PAA) that could deliver methylbenzene blue ammonium (MB), with a photosensitive antimicrobial agent. MB was then loaded onto ZIF-8-PAA nanoparticles, followed by the addition of AgNO_3_/dopamine to construct AgNPs. Finally, vancomycin/NH_2_-polyethylene glycol (Van/NH_2_-PEG) was used for a secondary modification to generate composite nanomaterials ZIF-8-PAA-MB@AgNPs@Van-PEG (ZPMAVP) (Fig. 8 a) [44]. This approach is more efficient in photodynamic treatment (PDT) for bacterial infections. The in vivo antibacterial assay using the New Zealand rabbit endophthalmitis model showed that PDT had better efficacy against *S. aureus* (Fig. 8 b) and MRSA (Fig. 8 c) compared to vancomycin (Van). This synergistic approach to chemotherapy and PDT for endophthalmitis is a promising strategy.

Cataract endophthalmitis is a common complication after cataract surgery, mainly caused by bacterial infection [45]. Cataract surgery may cause serious complications of endophthalmitis, such as corneal edema, spikes in intraocular pressure, posterior capsule opacification, cystoid macular edema, or even severe vision loss [46]. In order to improve the treatment effect of cataract endophthalmitis, Ye et al. created a new form of AuAgCu_2_O-bromfenac sodium nanoparticles (AuAgCu_2_O-BS NPs), which combines antibacterial and anti-inflammatory properties [43]. With the help of the photothermal effect, MRSA is destroyed by the release of metal ions (Ag^+^ and Cu^+^), and sodium bromfenac has antibacterial and anti-inflammatory properties while reducing the inflammatory response. In rabbit models of MRSA infection, the group treated with AuAgCu_2_O-BS NPs exhibited favorable anti-inflammatory and antibacterial effects with low toxicity and biosafety. It is worth mentioning that the nanosystem has no effect on intraocular pressure and no obvious toxicity, which has the potential for clinical application in the treatment of post-cataract endophthalmitis.

### 2.4 Other antibacterial strategies

There are other studies combining AgNPs with other substances to enhance the antimicrobial effect and thus be better used in the treatment of ocular infections. Ocular bandages differ from traditional medications such as eye drops with sustained release properties, to promote epithelial wound healing [47]. Yan et al. developed a novel kind of ocular bandage with poly (lactic acid) (PLA) electrospun nanofibrous membranes (EFMs), on which AgNPs are anchored [48]. Due to the fact that the AgNPs only adhere to the surface of PLA electrospun fibers, the inhibitory effect on *E. coli* and *S. aureus* can exceed 95 % at relatively low concentrations (0.72 mg/g). It can be seen that the antibacterial action of AgNPs enables the eye bandage to control infection. It is worth mentioning that the drug has both abilities to promote cell proliferation and antibacterial properties.

Due to foreign body irritation, long-term use of artificial eyes can easily lead to secondary infections. In order to avoid postoperative infections and to improve the antimicrobial properties of ocular prostheses, Yang et al. loaded AgNPs in artificial ocular prostheses, which greatly enhanced their antibacterial activity in vitro bacterial cultures [49]. Koev et al. deposited silver-doped Al_2_O_3_ nanolayers on glass ophthalmic prostheses to enhance the antibacterial properties of the prostheses and to prevent postoperative inflammatory reactions of the orbital contents [50]. The experimental results demonstrated that the nanocomposites have antimicrobial effects against both gram-positive and negative bacteria, which is very promising for ophthalmic implant prostheses to prevent ocular infections. In addition, Yee et al. doped the adhesive with AgNPs in eye tissue to improve the strength, fracture resistance, and antibacterial activity of the adhesive [51]. Guo et al. developed a core satellite-like bacterial-targeted Ag@CuPB nanosystem (ACPA), which can eliminate *S. aureus* infections by releasing metal particles and generating ROS, and demonstrated the effect in a diabetic mouse model of keratitis [52]. The therapeutic functions of ACPA realize the synergistic management of polymetallic ions and provide a strategy for chronic wound healing of diabetic infectious Keratitis. In summary, it can be seen that AgNPs have great potential for joint application with novel materials and drug delivery carriers due to their excellent antimicrobial properties.

## 3 Gold nanoparticles (AuNPs)

**Fig. 9:**
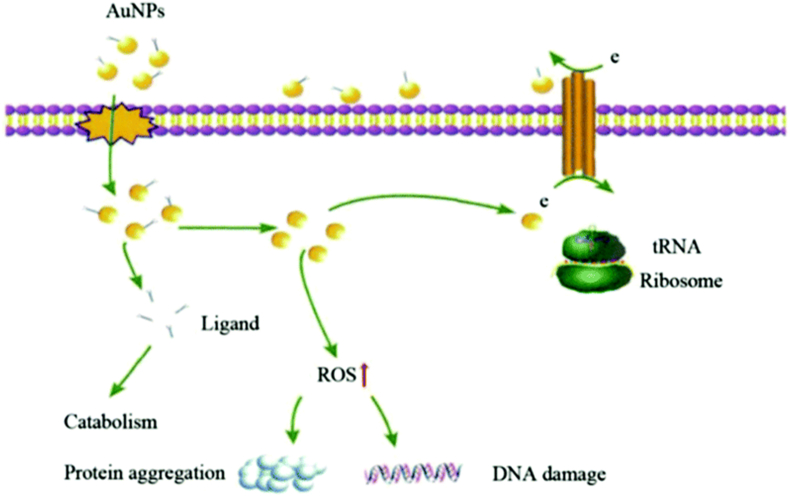
AuNPs' antibacterial mechanism [53]. Reproduced with permission. Copyright 2020 The Royal Society of Chemistry.

Studies have shown that the antimicrobial effect of AuNPs is primarily achieved by disrupting the bacterial cell membrane and entering the bacterial cell to affect cellular functions (Fig. 9) [[53], [54], [55]]. Researchers agree that these antimicrobial properties depend on certain physicochemical characteristics and the synthesis of AuQDs (nanoparticles with dimensions of 2–10 nm) exemplifies this principle (Nanoparticles with dimensions of 2–10 nm) is a good example [56]. Although AuNPs alone do not have naturally antibacterial like AgNPs, they can still be used as delivery systems to help treat drug-resistant bacteria by restoring the effects of antibiotics and synergizing with antibiotics to improve antibacterial effectiveness [57]. Al et al. prepared a gold nano-formulation of bexifloxacin (an antibiotic used for ocular infections) and used it synergistically with quercetin. They found that this approach had a significant inhibitory effect on the growth of *S. aureus*, aiming to provide new ideas for the treatment of ophthalmic infections [58].

**Fig. 10:**
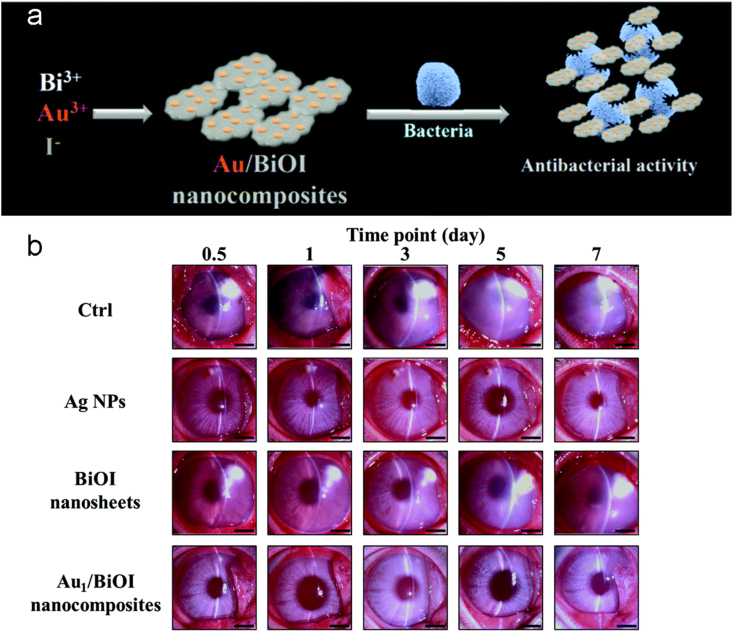
(a) Antimicrobial agent Au/BiOI nanocomposites synthesis. (b) Therapeutic slit-lamp plots of AgNPs, BiOI nanosheets, and Au_1_/BiOI nanocomposites in rabbit eyes with bacterial keratitis at different times after treatment [59]. Reproduced with permission. Copyright 2018 The Royal Society of Chemistry.

In another study, for rabbits with bacterial keratitis caused by *S. aureus*, catalytic gold-doped bismuth oxyiodide (Au/BiOI) nanocomposites were used to successfully alleviate the condition [59]. By in situ doping Au NPs and gold–iodide (Au–I) complexes into BiOI nanosheets, Hsu et al. have created Au/BiOI nanocomposites with strong oxidase-like activity (Fig. 10 a). Au_1_/BiOI nanocomposites are highly biocompatible and show favorable therapeutic effects against BK in in vivo experiments (Fig. 10 b).

**Fig. 11:**
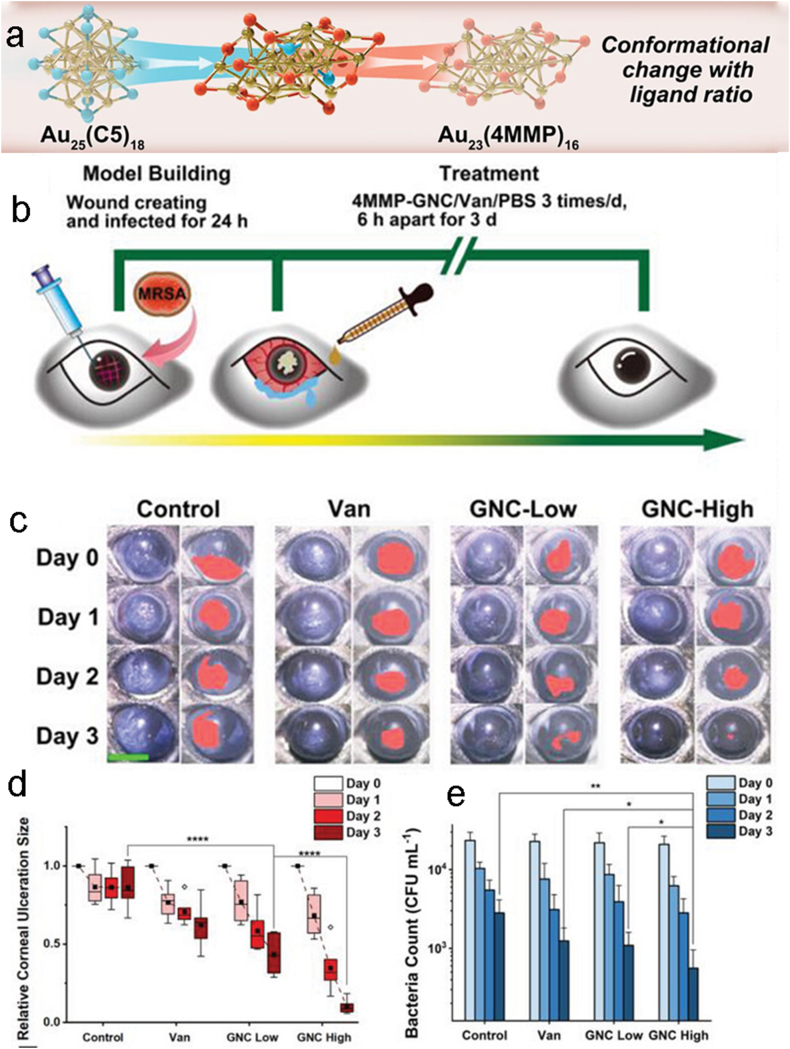
(a)The design idea of GNC. (b)The in vivo experimental procedure. (c)stereomicroscope pictures showing the various groups' levels of eye infections. (d)The size of the corneal ulcer in various treatment groups and at various times is shown statistically. (e)Results of the bacterial counts of swabs taken from each group's ocular secretions [60]. Reproduced with permission. Copyright 2023 Wiley-VCH GmbH.

Pang et al. enhanced the antibacterial effect of gold nanoclusters (GNCs) by combining two ligands (a thiol fragrance, 4-mercapto-4-methyl-2-pentanol [4MMP] and a thiolated zwitterionic ligand [C5]) [60]. By adjusting the proportion of ligands, they prepared 4MMP-GNC with the best antimicrobial effect (Fig. 11 a). To assess the efficacy of keratitis in vivo, they established a mouse model of keratitis with MRSR and treated the mice with PBS, Van, and two concentrations of 4MMP-GNC(16 and 64 μg*mL^−1^), for three days respectively (Fig. 11 b). The results showed that the ulcer area was significantly reduced in the 4MMP GNC-treated group, while the ulcer improvement was minimal in the negative control group (Fig. 11c and d). Both concentrations of 4MMP-GNC were effective in reducing the number of bacteria (Fig. 11 e). This method has a significant effect on keratitis in a short period. The positive effects of AuNPs antimicrobial can thus be seen. It is promising that the safety and sustainability of AuNPs are expected to be addressed with the in-depth study of their green synthesis methods as well as toxicology [61].

## 4 Gallium nanoparticles (GaNPs)

**Fig. 12:**
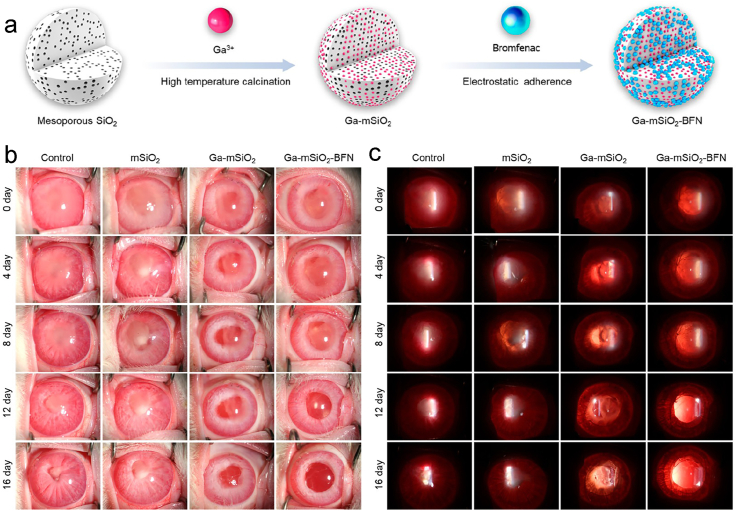
(a) Synthesis route of the Ga-mSiO_2_-BFN. (b) Representative micrographs of MDR-PA-infected PCE rabbits treated differently at various intervals of time using slit-lamp dispersion and (c) retro-illumination [66]. Reproduced with permission. Copyright 2022 American Chemical Society.

Unlike the common gold and silver nanoparticles, the antibacterial effect of gallium nanoparticles has been paid more and more attention [62]. Important metabolic processes such as cellular respiration, oxidative stress, and bacterial DNA synthesis are dysregulated and disrupted by gallium [63]. Therefore, Gallium has the effect of inhibiting bacterial growth, especially MRSA [64] and *P. aeruginosa* [65]. Qiao et al. prepared Ga-mSiO2-BFN by infiltrating gallium and bromfenac (BFN) into a Si-O-Si structure and developed a multifunctional nanosystem that combines the antimicrobial effect of Ga with the anti-inflammatory effect of BFN to synergistically treat post-cataract endophthalmitis (Fig. 12 a) [66]. They created rabbit models of MDR-PAinfected PCE and conducted controlled experiments to more accurately evaluate in vivo effectiveness. As can be observed, on the 16th day following treatment, the effect of the Ga-mSiO_2_-BFN group was significantly better than the other groups, and the infection and inflammation were satisfactorily controlled (Fig. 12b and c). This nanosystem has advantages over conventional drugs because of its low drug resistance potential as well as dual anti-inflammatory and antimicrobial effects.

## 5 Zinc nanoparticles and copper nanoparticles

**Fig. 13:**
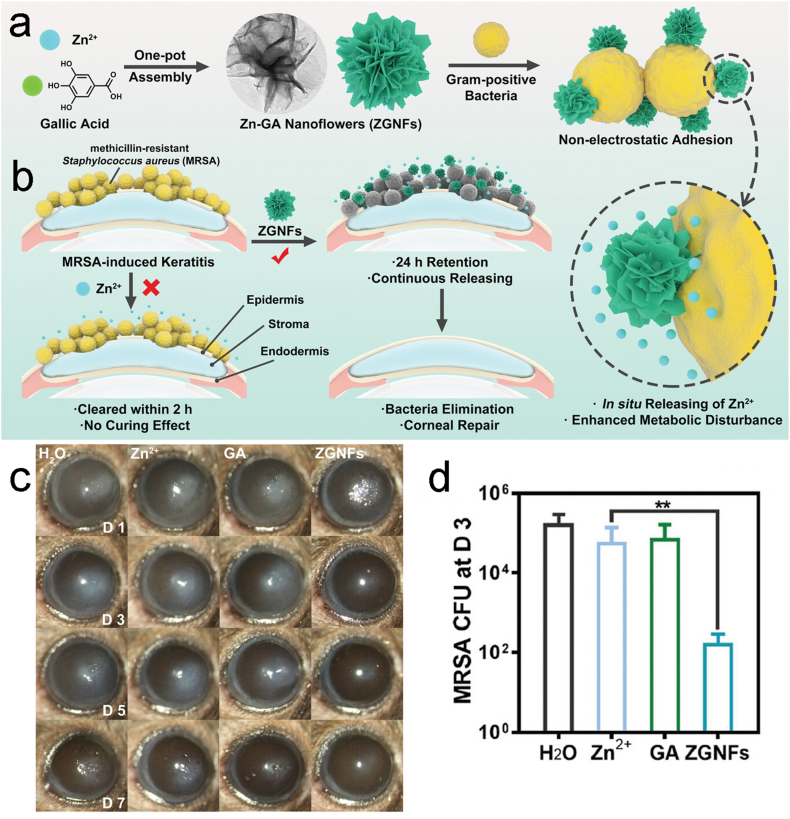
(a)The preparation routine of ZGNFs and (b)how interact with gram-positive bacteria. (c)Slit-lamp micrographs of mouse eyeballs treated in a variety of ways over one to seven days. (d)MRSA CFU counting in the corneas on day three after various treatments [69]. Reproduced with permission. Copyright 2023 Wiley-VCH GmbH.

The antimicrobial properties of pure zinc are not sufficient to combat biofilms and germs that are resistant to antibiotics [67], so the antimicrobial properties of Znic are commonly found in complexes. The production of Zn ions and the generation of reactive oxygen species may be the bactericidal mechanism of ZnO and the bactericidal effect is related to its size [68]. Huang et al. used a simple one-pot procedure to create Zn^2+^-gallic acid nanoflowers (ZGNFs) (Fig. 13 a), which are particularly able to adhere to Gram-positive bacteria and used to treat MRSA-induced bacterial keratitis [69]. Compared to free Zn^2+^, ZGNFs have excellent bactericidal ability in complex environments such as protein solutions, which is attributed to their special adhesion and in-situ release of Zn^2+^ characteristics (Fig. 13 b). As shown in slit-lamp microscope images, the MRSA-infected keratitis model exhibits typical clinical symptoms such as turbidity and suppuration (Fig. 13 c). On the third day after ZGNF administration, the therapeutic effect was significant and most of the MRSA was eliminated (Fig. 13 d). Because of their exceptional bactericidal activity and superior biocompatibility, ZGNFs have great application prospects in the treatment of keratitis caused by Gram-positive bacteria.

ZnONPs possess attractive antimicrobial properties due to their small particle size and larger specific surface area, which are mainly realized by releasing reactive oxygen species [70]. Additionally, the photocatalytic activity [71] and biosafety [70] of ZnONP have been confirmed. El-Gendy et al. synthesized ZnONPs using laser ablation and conducted studies to demonstrate its effect on multidrug-resistant ocular pathogens such as MRSA and *P*. *aeruginosa*, providing new ideas for antioxidant drug carriers for the treatment of retinal diseases [72]. This study showed that when exposed to 400 nm fs laser irradiation, ZnONPs were biocompatible with retinal epithelial cells (ARPE-19) and significantly inhibited bacterial growth. But before clinical use, more in vivo studies are needed to rule out adverse consequences. Hoyo et al. embedded three compounds, ZnO NPs, chitosan (CS) and gallic acid (GA) onto the CL [73]. The CS/GA/ZnO hybrid coating not only exhibited high antimicrobial properties against *S. aureus*, but also greatly improved the surface wettability of the CL, increased the comfort of the wearer, and minimized the problems associated with CL abrasion.

The usual valences of Cu, a redox element, are +2 or +1. Therefore, redox-active Cu ions may potentially be implicated in electron-transfer reactions, in contrast with zinc and silver [74]. The antimicrobial mechanism of copper has been mainly described as direct disruption of cell membranes [75], oxidative damage caused through redox reactions [76], and NP-mediated dissipation of cell membrane potential [77]. Copper oxide nanoparticles (CuONP) have strong antimicrobial activity against a variety of pathogenic microorganisms [78], which has aroused people's interest in their antibacterial applications in ophthalmology. Through in vitro experiments, Tuby et al. loaded zinc-doped copper oxide nanocoatings on the surface of contact lenses and that the adhesion of *S*. *epidermidis* and *P. aeruginosa* to contact lenses decreased, indicating that the Zn CuO nanocomposites attached to contact lenses have antibacterial properties [79]. Similar to this study, Nahum et al. loaded Zn-CuO onto contact lenses using an acoustochemical coating technique and determined that it had effective antibacterial activity against *S*. *epidermidis* and *P*. *aeruginosa* [80]. Kharaghani et al. also attached CuNPs to polyvinyl alcohol contact lenses and demonstrated good antimicrobial activity with no toxicity to mammalian cells [81]. Although the three studies confirmed antimicrobial properties, they were limited to in vitro studies, and therefore require further in vivo studies to evaluate the value of the clinical application.

## 6 Molybdenum (Mo) and Titanium (Ti)

The development of molybdenum-based nanomaterials in a variety of biological applications is due to their unique physicochemical features, particularly their antibacterial activity and comparatively low toxicity [82]. The antimicrobial strategy of molybdenum-based nanomaterials involves three main mechanisms, including membrane disruption, oxidative stress, and binding to intracellular components [82]. Significantly, it has low toxicity to human cells. Li et al. used ion irradiation to customize transition metal sulfide (XS_2_, X = Mo/W) quantum dots for the treatment of bacterial keratitis and demonstrated their potential therapeutic effects on clinically resistant BK through in vivo experiments in rats [83]. In further study, Two-dimensional transition metal disulfide (MoS_2_) nanosheets were modified to include nanocavities to prevent *S. aureus* infection and damage to its biofilm [84,85]. Compared with traditional antibiotics and other antibacterial nanomaterials, they enhance biocompatibility and demonstrate excellent therapeutic ability in rat keratitis models. This approach offers the advantages of ease of manufacture, superior biocompatibility, and no resistance compared to typical antibiotics.

**Fig. 14:**
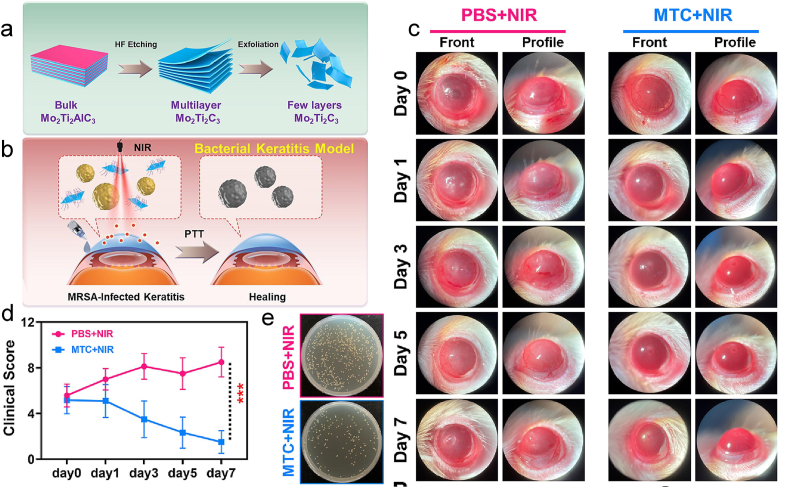
(a)The preparation of Mo_2_Ti_2_C_3_ and (b)the Schematic representation of the treatment of bacterial keratitis. (c) Typical slit-lamp micrograph appearance of MRSA-infected keratitis in mice administered 300 μg mL−1 eye drops (10 μL) at several observation time points using different methodologies. (d) Clinical scores of experimental rats at different time points for the condition of keratitis. (e)Bacterial culture plates of corneal tissue after seven days of treatment in both groups [85]. Reproduced with permission. Copyright 2023 Elsevier Ltd.

For the antimicrobial mechanism of Ti, it has been mentioned that TiO_2_ NPs absorb light and generate electron-hole pairs, which react with airborne H_2_O on the surface of nitrogen oxides to produce ROS [86]. This phenomenon is primarily attributable to cellular responses to osmotic stress, metabolism of cell envelope components, and uptake/metabolism of endogenous and exogenous compounds [87]. Hua et al. constructed MTC MXenes using two-dimensional transition metal carbides (MXenes) and photothermal nano reagent Mo_2_Ti_2_C_3_ (MTC) and demonstrated that MTC has an efficient killing effect on MRSA (Fig. 14a and b) [85]. In a mouse model of bacterial keratitis, the excellent photothermal conversion efficiency of MTC and NIR were used for synergistic treatment. Features such as corneal clouding on slit-lamp view indicate that the modeling was successful (Fig. 14 c). After 7 days of treatment, the clinical scores of the rats in the MTC + NIR group gradually declined (Fig. 14c), at which point the bacterial culture plates of their corneal tissue had significantly fewer bacteria than those of the control group (Fig. 14 e). It can be seen that MTC has the potential to enhance NIR therapy, which can then be further applied to bacterial keratitis.

## 7 Platinum nanoparticles (PtNPs)

The size of Pt NPs has an effect on their antimicrobial toxicity [88] and a strong negative zeta potential enhances the antimicrobial activity [89]. Its toxicity can be controlled or reduced by controlling the size of Pt NPs during the preparation process [90]. Using a new Pt (II) metallacycle created by Xu, the more precise second near-infrared (NIR-II) fluorescence imaging was accomplished and photo-activated tissue sterilization was improved under full laser irradiation [91]. In a range of animal models, including mice with keratitis, Pt1110 was shown to be efficient in accurate fluorescence imaging-guided photo-induced sterilization with little synergistic damage. In vivo experiment, NIR-II fluoresce imaging was carried out following the intravenous injection of Pt1110 nanoparticles (60 μM, 200 μL). Photographs taken 12 days later showed that the treatment effect of Pt1110 NPs combined with the laser group was better. Although ocular antimicrobial studies on PtNPs are not mature enough, it offers novel ideas for keratitis treatment.

## 8 Future perspectives and conclusions

Early treatment of bacterial infections of the eye is extremely important, timely and accurate intervention can help avoid serious damage such as blindness. Today's commonly used medicines such as eye drops need to develop new strategies to meet the challenges of ophthalmic antimicrobials due to low bioavailability and other reasons. With the rapid advancement of nanotechnology, extensive research has been conducted on the application of metal nanoparticles in antibacterial drugs. In this review, we reported the recent research progress of metal nanoparticles for ocular antimicrobial applications. Through the assembly and refinement of metal nanoparticles by various techniques, they have efficient, safe, and controllable ocular antimicrobial properties, which provide new strategies and ways to solve common ophthalmic diseases such as ocular infections and inflammations. Further research is also underway to address the environmental benefits as well as the cost-effectiveness of metal nanoparticles [[92], [93], [94]]. The environmental benefits and cost-effectiveness of metal nanoparticles are expected to be enhanced.

Nevertheless, studies have shown that the antimicrobial mechanism of metal nanoparticles is somewhat controversial [57] and metal nanoparticles can cause damage to the eyes under certain conditions [95]. Metal nanoparticles still face challenges and obstacles in the application of ocular antibacterial agents. These challenges can be categorized into some main areas: biocompatibility, toxicity, stability, and delivery, while there are some possible solutions to these challenges. For example, coating NPs with biocompatible materials such as polyethylene glycol (PEG) can reduce cytotoxicity and improve biocompatibility [96]. Delivery systems like liposomes, lipid NPs, polysaccharide NPs, and polymeric NPs can control the release of NPs, thereby minimizing peak concentrations and reducing toxicity [35]. Utilizing advanced delivery systems such as nanoparticle-loaded contact lenses helps improve NPs retention and penetration in ocular tissues [97].

Therefore, it is necessary to understand the activity patterns of metal nanoparticles more accurately and sufficiently to fully utilize their antimicrobial potential and choose appropriate strategies to synthesize nanomedicines that are non-toxic to the eye. In the future, it is expected to carry out research in more aspects, such as strengthening basic research related to the antimicrobial mechanism of metal nanoparticles, developing more types and functions of metal nanoparticles, and exploring more scenarios and forms of metal nanoparticles that can be used for ocular antimicrobials. In addition, due to the development of nanomedicine is not mature enough, more effective regulatory measures are needed to regulate the research and use of nanomedicine. We hope that better ophthalmic antibacterial treatment strategies can be utilized. And we believe that metal nanoparticles have the potential to make a significant impact and contribution to ophthalmology and human health.

## CRediT authorship contribution statement

**Mingyou Zhang:** Writing – original draft. **Yuhang Cheng:** Writing – original draft. **Hongjin Li:** Writing – review & editing, Conceptualization. **Mengdie Li:** Writing – review & editing. **Qixiang Yang:** Resources, Investigation. **Kaifang Hua:** Writing – review & editing. **Xiaofei Wen:** Writing – review & editing. **Yun Han:** Writing – review & editing. **Gang Liu:** Writing – review & editing, Conceptualization. **Chengchao Chu:** Writing – review & editing, Conceptualization.

## Declaration of competing interest

The authors declare that they have no known competing financial interests or personal relationships that could have appeared to influence the work reported in this paper.

## Data Availability

No data was used for the research described in the article.
